# Impact of psychotic symptoms on clinical outcomes in delirium

**DOI:** 10.1371/journal.pone.0200538

**Published:** 2018-07-13

**Authors:** Soo-Hyun Paik, Joung-Sook Ahn, Seongho Min, Ki-Chang Park, Min-Hyuk Kim

**Affiliations:** 1 Bucheon Ki Hospital, Bucheon, South Korea; 2 Department of Psychiatry, Yonsei University Wonju College of Medicine, Wonju, South Korea; Department of Psychiatry and Neuropsychology, Maastricht University Medical Center, NETHERLANDS

## Abstract

Delirium is an acute disturbance in attention and awareness in response to one or more physiological stressors that is closely related to poor clinical outcomes. The aim of this study is to investigate whether delirium patients with psychotic symptoms (PS) would have unique clinical characteristics and outcomes. A retrospective chart review was performed on the patients with delirium due to general medical conditions to assess clinical characteristics and outcomes. All patients were assessed by Delirium Rating Scale-revised-98 and classified as having PS when scored two or more on at least one of the psychotic symptom items (perceptual disturbances, delusions, and thought process abnormalities). Of 233 patients with delirium, 116 (49.8%) manifested PS. Patients with PS were younger, more likely to use antipsychotics to manage delirium, and had more hyperactive motor subtype than patients without PS. Logistic regression analysis showed that odds ratio of psychotic symptoms for having in-hospital mortality was 0.27 (95% CI = 0.08–0.94) after controlling age, sex, disease severity, comorbidity, number of medications, etiologies, motor subtypes, delirium severity and use of antipsychotics. The present study demonstrated that PS of delirium was associated with unique clinical characteristics and may affect the clinical course in a psychiatry-referral sample.

## Introduction

Delirium is an acute disturbance in attention and awareness in response to one or more physiological stressors frequently encountered in hospital settings[1]. Delirium in critically ill patients is associated with higher morbidity and mortality, such as longer duration of mechanical ventilation, longer length of stay in ICU and hospital, and higher probability of transfer to medical institutions [2]. Though delirium is primarily known as a disorder of cognition, the non-cognitive symptoms are frequently encountered [3], and possibly influence the course of delirium. Previous studies have mainly focused on the role of motor symptoms of delirium on clinical outcomes [4, 5], and little is known about the impact of non-cognitive, non-motor symptoms of delirium.

Psychotic symptoms are frequently observed among dementia patients and often associated with poor clinical outcome [6]. In particular, delusions and hallucinations are associated with increased risk of cognitive and functional decline [7], and faster functional impairment and increased mortality risk [6, 8, 9]. Likewise, delusions and hallucinations are frequently reported among delirium patients. More than 40% of delirious patients have delusions and hallucinations [3, 10, 11]. Patients with psychotic symptoms of delirium display psychomotor agitations and aggressive behaviors [3, 12], more often than those without psychotic symptoms, and may lead to self-harm or noncompliance that would lead to medical complications and poor clinical outcomes. However, the role of psychotic symptoms in delirium has not been sufficiently investigated yet. This study was designed to investigate the clinical characteristics and outcomes of delirium with psychotic symptoms.

## Materials and methods

### Subjects and procedure

The study was carried out in a university hospital in South Korea. The authors reviewed a series of patients referred to adult and elderly psychiatric consultation-liason services from January to June 2012. Among total of 1147 patients who were referred to psychiatric consultation services, we identified 353 (30.8%) patients diagnosed as delirium due to general medical conditions according to Diagnostic and Statistical Manual of Mental Disorders, 4^th^ edition (DSM-IV) by trained psychiatrists. Subjects with delirium related to alcohol withdrawal and superimposed on dementia (n = 23), which was diagnosed before the admission to the hospital, and with insufficient medical records or DRS-R-98 scores (n = 97) were excluded and 233 patients were included in the final analyses. There were no differences in age, sex, disease severity, comorbidity, number of medication, etiology of delirium, psychiatric history, length of hospital stay between subjects with full DRS-R-98 scores and those without. The mean age was 71.1 years old (SD = 13.22) and 163 (70%) subjects were males. Mean APACHE-II score and age-adjusted CCI were 12.38 (SD = 4.485) and 5.46 (SD = 2.877) respectively.

The protocol and conduct of this retrospective study was approved by the Institutional Review Board of Yonsei University Wonju College of Medicine (IRB No. YWMR-14-5-050) before this study was conducted. A waiver of informed consent was obtained as this study was a retrospective review of medical records.

### Assessments

A structured case report form was filled for each patient to collect the following demographic and clinical data; age, sex, use of psychotropic medications that would influence the course of delirium (opioids, psychostimulants, antidepressant, benzodiazepines, and hypnotics), previous psychiatric diagnoses, and use of antipsychotics for the management of delirium symptoms. Charlson Comorbidity Index (CCI) was calculated to assess comorbidity burden. CCI has good reliability and excellent correlation with mortality, disability, readmission, and length of stay outcomes [13]. Since age-adjusted CCI was an independent prognostic factor of mortality after adjusting other covariates [14], we assessed age-adjusted CCI based on the reviewed chart. Acute Physiology and Chronic Health Evaluation II (APACHE-II) scoring system was used to evaluate the disease severity, based on lab findings and medical records within one day before referral to psychiatric consultation. APACHE-II takes the patient’s age and 12 physiological measurements into account and has good predictive value for acutely ill patients [15, 16], Attribution of etiology was made according to a standardized Delirium Etiology Checklist (DEC) with 12 categories [17]. The presence of multiple potential causes of delirium was also documented for each case without rating.

A Delirium Rating Scale-revised-98 (DRS-R-98) was conducted by a trained psychiatrist at the first visit. DRS-R-98 is a 16-item clinician-rated scale to diagnose and assess the severity of delirium [18]. It is composed of 13 severity items (sleep wake cycle, perceptual disturbance/hallucination, delusions, lability of affect, language, thought process abnormality, motor agitation, motor retardation, orientation, attention, short-term memory, long-term memory, and visuospatial ability) and three diagnostic items (temporal onset of symptoms, fluctuation, and physical disorder). Each item is rated from 0 (absent/normal) to 3 (severe) and higher scores indicate more severe symptom severity. The Korean version of the DRS-R-98 scale has been demonstrated to be valid and reliable for assessing delirium as well as discriminating delirium from dementia [19]. According to Meagher and colleagues [3], psychotic symptoms (PS) were defined as having two or more scores on any one of three items from DRS-R-98 psychotic symptom (perceptual disturbances/hallucination, delusions and thought process abnormalities). Motor subtype was classified by the definition proposed by Lipowski [20–23]: (1) hyperactive subtype is characterized as restlessness, agitation, hyperactivity and aggressiveness; (2) hypoactive subtype as decreased activity and reactivity, retardation, lethargy and drowsiness; and (3) mixed subtype as shifts between hypoactivity to hyperactivity [23]. Though Lipowski’s description is not a validated scale, this has moderate to high degree of consensus (55 to 76%) with other methods of defining motor subtype of delirium [24].

The following clinical outcome measures were collected; length of hospital stay, need of Intensive Care Unit (ICU) care, length of ICU stay, need of mechanical ventilation (MV) and duration of MV, in-hospital mortality, accidents due to delirium, and transfer to medical institutions. Accidents due to delirium were defined as either of accidental tube removal by patients themselves, fall down injuries, or other mishaps due to delirium symptoms.

### Statistical analysis

χ^2^-tests and independent t-tests were performed to compare the clinical characteristics, delirium symptoms and outcomes between delirium with psychotic symptoms group (PS group) and those without (non-PS group). Mann-Whitney U tests were used for the non-parametric continuous variables. Pearson correlation analyses were used to examine the association between psychotic symptoms and other symptoms of delirium. Logistic regression and multiple regression analyses were conducted to investigate the association between PS and clinical outcomes (In-hospital mortality, accidents due to delirium, and length of hospital and ICU stay). All statistical analyses were performed by SPSS 18.0 (SPSS Inc., Chicago, IL, USA).

## Results

### Differences in clinical characteristics and outcomes

Of 233 patients, 116 (49.8%) patients had ≥2 scores on any one of psychotic symptoms items. Among them, 103 (44.2%) had perceptual disturbance, 34 (14.6%) had delusions, and 33 (14.2%) had thought process abnormalities. Fifty three (23.2%) patients scored ≥2 on two of three psychotic symptoms items and only six (2.7%) rated two or more on all the three psychotic symptoms items. Fifty seven (24.5%) patients scored ≥3 on any one of three psychotic symptoms items. Pearson correlation analyses revealed that there were no associations between any of the three psychotic symptoms in patients with psychotic symptoms.

Patients with psychotic symptoms were younger than patients (PS: 68.9±14.5 vs. non-PS: 73.2±11.5, χ^2^ = 2.487, *p* = .014), manifested more hyperactive motor subtype (PS: 62.1% vs. non-PS group: 42.7%, χ^2^ = 10.218, *p* = 0.006), and used antipsychotics to manage delirium symptoms more frequently than those without psychotic symptoms (PS: 80.2% vs. non-PS: 61.5%, χ^2^ = 9.786, *p* = .002) (Table 1).

**Table 1:** Comparisons of demographic and clinical characteristics.

	With psychotic symptoms (n = 116)	Without psychotic symptoms (n = 117)	χ^2^/t
**Age (mean±SD)**	**68.93±14.5**	**73.20±11.5**	2.487*
Sex (male)	82 (70.7%)	80 (68.4%)	0.147
APACHE-II	12.40±4.76	12.37±4.21	-0.049
CCI (age adjusted) (mean±SD)	5.27±2.84	5.66±2.91	1.037
Number of medications (mean±SD)	11.08±4.39	10.89±4.39	-0.328
Number of etiologies (mean±SD)	1.54±0.66	1.51±0.69	-0.341
Presence of multiple etiologies	53 (45.7%)	48 (41.0%)	0.516
**Motor subtype**			
** Hyperactive**	**73 (62.9%)**	**50 (42.7%)**	10.218**
** Hypoactive**	**2 (1.7%)**	**6 (5.1%)**	
** Mixed**	**41 (35.3%)**	**61 (52.1%)**	
Psychiatric comorbidities	27 (23.3%)	23 (19.7%)	0.452
Use of psychotropic agents	13 (11.2%)	17 (14.5%)	0.573
**Use of antipsychotics for delirium**	**93 (80.2%)**	**72 (61.5%)**	9.786**

APACHE-II; Acute Physiology and Chronic Health Evaluation II, CCI; Charlson Comorbidity Index.

* p < .05,

** p < .005.

The two groups showed no significant differences in sex distribution, mean APACHE-II and CCI score, numbers of medications and etiologies, presence of psychiatric comorbidities and use of antipsychotics. The proportions of patients referred from medical department (PS: 70.7% vs. non-PS: 62.4%, χ^2^ = 1.116, *p* = .291) and those with psychiatric illness (PS: 23.3% vs. non-PS: 19.7%, χ^2^ = .452, *p* = .501) were comparable between groups. The distributions of etiology and comorbid psychiatric illness were not different between groups as well; in whole sample, the most common etiologies of delirium according to DEC were systemic infection (n = 81, 36.3%), followed by metabolic and endocrine abnormalities (n = 55, 24.6%) and organ insufficiency (n = 54, 24.2%). The frequency of systemic infection was 40 (34.5%) in the PS group and 41 (35.0%) in the non-PS group (χ^2^ = .008, *p* = .928). The most common psychiatric comorbidity was mood disorder (n = 20, 8.9%), followed by substance use disorder (n = 15, 6.7%).

Table 2 showed differences in clinical outcomes between groups. Delirium with psychotic symptoms was associated with lower incidence of in-hospital mortality (PS: 4.3% vs. non-PS: 12.0%, χ^2^ = 4.558, *p* = 0.033). Other clinical outcomes were not significantly different between groups.

**Table 2:** Comparisons of clinical outcomes.

	With psychotic symptoms (n = 116)	Without psychotic symptoms (n = 117)	χ^2^/U
Length of hospital stay (days) ^a^	20.50 (13, 36.75)	21.00 (11, 44)	6622.000
ICU care	54 (46.6%)	54 (46.2%)	0.004
Length of ICU stay (days) ^a^	7.50 (4, 15)	8.50 (5, 19.25)	1292.500
**In-hospital mortality**	**5 (4.3%)**	**14 (12.0%)**	4.558*
Mechanical ventilation	23 (19.8%)	31 (26.5%)	1.455
Duration of mechanical ventilation (days) ^a^	6.00 (3, 8)	6.00 (3, 22)	312.000
Accidents due to delirium	46 (39.7%)	48 (41.0%)	0.045
Transfer to medical institutions	43 (37.1%)	35 (29.9%)	1.339

ICU; Intensive Care Unit.

^a^ Median (Interquantile range; IQR)

* *p* < .05.

### Differences in clinical outcomes

Tables 3 and 4 showed the results of logistic and multiple regression analyses. In univariate analyses, in-hospital mortality was lower in patients with PS (OR = 0.33, *p* = .040) and higher in patients with higher APACHE-II (OR = 1.14, p = .011) and CCI scores (OR = 1.27, *p* = .009), higher number of etiologies (OR = 1.90, *p* = .039) and mixed motor subtype (OR = 6.90, *p* = .003). Multivariate logistic regression revealed that absence of psychotic symptoms (OR = .027, *p* = .039), higher APACHE-II score (OR = 1.16, *p* = .020) and mixed motor subtype (OR = 10.03, *p* = .003) predicted in-hospital mortality. When redefining psychotic symptom as having either of hallucination or delusion (not including thought process abnormality) to confirm this association in patients with core psychotic symptoms, the result was comparable to original results (adjusted OR = 0.28, 95%CI = 0.08–0.97, *p* = 0.045). Cox regression analysis, which was performed to examine the role of the heterogeneous nature of subjects or time effect, on patients referred from the medical department revealed similar findings (adjusted HR = 0.15, 95% CI = 0.30–0.78, *p* = 0.024). A logistic regression analysis to investigate the prediction of accident due to delirium found only age (OR = 1.03, *p* = .018), male gender (OR = 2.38, *p* = .020) and mixed motor subtype (OR = 0.10, *p* < .001) as meaningful predictors.

**Table 3:** Logistic regression analysis for in-hospital mortality and accidents.

	In-hospital mortality		Accidents due to delirium^b^	
	OR (95% CI)	aOR (95% CI)	OR (95% CI)	aOR (95% CI)
Age	1.00 (0.97–1.04)	0.96 (0.91–1.02)	1.01 (0.99–1.03)	1.03 (1.01–1.06)*
Sex (male)	1.06 (0.39–2.91)	2.06 (0.57–7.43)	2.36 (1.28–4.34)*	2.38 (1.15–4.94)*
APACHE-II	1.14 (1.03–1.26)*	1.16 (1.02–1.31)*	0.995 (.94–1.06)	0.97 (0.89–1.04)
CCI (age adjusted)	1.27 (1.06–1.52)*	1.209 (0.98–1.50)	1.03 (0.94–1.12)	1.04 (0.92–1.17)
Number of medications	1.07 (0.96–1.19)	1.09 (0.96–1.23)	1.00 (0.94–1.06)	0.98 (0.91–1.05)
Number of etiologies	1.90 (1.03–3.49)*	1.89 (0.92–3.88)	0.90 (0.61–1.33)	0.98 (0.60–1.60)
Motor subtype				
Hyperactive	Reference	Reference	Reference	Reference
Hypoactive	5.71 (0.53–62.24)	2.95 (0.20–42.94)	0.21 (0.04–1.06)	0.15 (0.03–0.90)
Mixed	6.90 (1.94–24.56)**	10.03 (2.17–46.39)**	0.12 (0.06–0.22)**	0.10 (0.05–0.21)**
DRS-R-98^a^	0.95 (0.87–1.05)	1.08 (0.94–1.23)	1.07 (1.01–1.12)	1.01 (0.94–1.09)
Use of antipsychotics	0.54 (0.21–1.40)	0.65 (0.20–2.14)	2.37 (1.28–4.41)	1.42 (0.65–3.10)
Psychotic symptoms	0.33 (0.12–0.95)*	0.27 (0.08–0.94)*	0.95 (0.56–1.60)	0.58 (0.29–1.16)

^a^ Sum of DRS-R-98 severity items except perceptual disturbance, delusion and thought process abnormalities

^b^ Accidents due to delirium were defined as either of accidental tube removal by patients themselves, fall down injuries, or other mishaps due to delirium symptoms.

* *p* < .05,

** *p* < .005.

**Table 4:** Predicting factors for length of stay in hospital and ICU.

	Length of hospital stay^a^		Length of ICU stay^a^	
	B	Β	B	β
Age	**-0.016**	-0.241**	**-0.020**	-0.348**
Sex (male)	-0.025	-0.014	-0.016	-0.008
APACHE-II	-0.008	-0.042	0.032	0.172
CCI (age adjusted)	-0.031	-0.103	-0.002	-0.006
Number of medications	**0.059**	0.299**	0.042	0.195
Number of etiologies	-0.150	-0.118	0.030	0.026
Motor subtype				
** **Hyperactive	Reference	Reference	Reference	Reference
** **Hypoactive	-0.017	-0.004	0.203	-0.033
** **Mixed	0.082	0.047	0.264	0.152
DRS-R-98^b^	0.006	0.036	0.026	0.172
Use of antipsychotics	-0.022	-0.012	0.230	0.127
Psychotic symptoms	-0.174	-0.101	-0.338	-0.201

^a^ Transformed to ln scale

^b^ Sum of DRS-R-98 severity items except perceptual disturbance, delusion and thought process abnormalities

** *p* < .005.

Multiple regression analyses revealed that psychotic symptoms were not associated with length of hospital and ICU stay (Table 4). Age (B = -0.016, *p* = .001) and number of medication (B = 0.059, *p* = .000) predicted length of hospital stay while only age (B = -.0.020, *p* = .004) predicted length of ICU stay.

## Discussion

We investigated the differences in clinical characteristics and outcomes between psychotic and non-psychotic delirium. To the best of our knowledge, this is the first study to investigate the association between psychotic symptoms of delirium and clinical outcomes.

The prevalence of psychotic symptoms in our study was 49.8%, comparable to previous findings ranging from 31% to 49% [3, 10, 11]. Among psychotic patients, delusion and hallucination were not associated with cognitive symptoms, implying that these symptoms may not result from the misunderstanding of the external environment due to impaired cognitive functions [3]. In addition, psychotic symptoms in delirium were not associated with comorbid psychiatric conditions. These findings suggest the pathophysiology of psychotic symptoms of delirium may be different from that of functional psychotic illness, such as bipolar disorder and schizophrenia [3, 25]. This study showed no difference in the past history of psychiatric illness psychotic and non-psychotic delirium patients. This supports previous findings that the pathophysiological causes of psychotic symptoms in bipolar disorder, schizophrenia or functional psychotic illness are different from those found in delirium [3, 10]. The results were consistent with previous studies that hyperactive delirium referred for psychiatric consultation was more likely to involve psychotic symptoms [11, 26]. Meagher and colleagues, on the contrary, showed no association between motor subtype and psychotic symptoms [3]. Methodological differences including sample inclusion and classification may account for the mixed results.

An interesting result was observed in terms of motor subtypes that mixed subtypes affect accidents and in-hospital mortality in a different way. Additional analysis to investigate the role of motor subtypes on in-hospital mortality after controlling for confounding factors and accidents showed comparable results. This suggests that motor subtype may serve as a risk factor for in-hospital mortality while as a protective factor for delirium-associated accidents and that the effects of motor subtype on in-hospital mortality may not be mediated by accidents.

In this study, the logistic regression revealed the odds ratio of the presence of psychotic symptoms for having in-hospital mortality as 0.27 (95% CI = 0.08–0.94) after controlling for the critical confounding factors, such as disease severity, comorbidity, number of medications, number of etiologies, motor subtype, delirium severity, and use of antipsychotics. This result should be interpreted carefully since only a small number of in-hospital death was observed in this study. Despite, here we deliberately suggest some explanations for this result. Firstly, psychotic symptoms may serve as ‘warning signs’ of the poor physical condition, or vice versa. Psychotic symptoms often induce agitation or inappropriate behavior and may lead to draw more clinical attention and eventually reducing mortality. Inversely, poor medical condition itself can be a predisposing factor for psychotic symptoms. Second can be that psychotic symptoms would play an independent role on the clinical course of delirium patients. In this study, psychotic symptoms were associated with in-hospital mortality, but not with delirium-associated with accidents. Further studies with larger sample size with prospective design would be necessary to verify the results. Third can be the unique nature of psychotic symptoms manifested in delirium patients. Unlikely to dementia, which is a neurodegenerative disorder, delirium is caused by physiological disturbance and is considered to be reversible. Thus the underlying neurophathophysiology of psychotic symptoms manifested in delirium and dementia may be associated with different disease courses [3].

This study has several strengths. First, whole assessment was done by trained psychiatrists using standardized tools such as DSM-IV and DRS-R-98 based on the direct observation. Furthermore, excluding preexisting dementia and alcohol withdrawal delirium would avoid confounding effects of psychotic symptoms from other causes and strengthen the association between psychotic symptoms and clinical outcomes in delirium caused by general medical conditions. Secondly, our sample included patients with a broad range of etiologies and medical or surgical conditions, with no etiologic differences between psychotic and non-psychotic delirium. This may broaden the generalizability beyond delirium occurred in specific populations. At the same time, the heterogeneity of the sample would affect the clinical outcomes and obscure our result at the same time. Results from two subgroup analyses, first including only patients over 65 years of age and second only medical patients were comparable to the results of the whole sample.

Despite these considerable strengths, there are also limitations that should be noted. First, the study design was retrospective and the number of sample was relatively small. Second, this study involved only patients referred for psychiatric consultation and those fully assessed with DRS-R-98. Though no differences in clinical characteristics and outcomes between subjects with full DRS-R-98 scores and those without were observed, the sampling methods may lead to an overrepresentation of delirium with particular problems. Third, the assessment of psychotic symptoms based on DRS-R-98 was done at a single point in time. Because symptoms and severity of delirium fluctuate, assessments at multiple points would likely provide more reliable information. Meanwhile, according to Meagher and colleagues, the pattern of delirium symptoms does not change over time in specific DRS items [27]. Fourth, since the direct causes of death were not determined in this study, the direct causality between the physical conditions that led to delirium and mortality is difficult to define. Fifth, we assessed motor subtype based on clinical data, not using validated tools such as Delirium Motor Subtyping Scale (DMSS) [24], which would underestimate the effect of motor subtype in clinical outcomes. However, Lipowski first introduced the concept of hyperactive, hypoactive and mixed variants of delirium and this concept has been widely used in clinical situations as well as in researches [23, 24, 28]. Thus we considered Lipowski’s concept would have good guiding properties for determining motor subtype of delirium. Lastly, there is a possibility of the inclusion of undiagnosed dementia since we excluded only diagnosed dementia prior to the admission.

In conclusion, we demonstrated that psychotic symptoms in delirium were associated with unique clinical characteristics and outcomes and may serve as a risk factor of adverse outcome in delirious patients referred for psychiatric consultations. Further prospective large study would be necessary to validate the role of psychotic symptoms in clinical outcomes.
